# Using data from food challenges to inform management of consumers with food allergy: A systematic review with individual participant data meta-analysis

**DOI:** 10.1016/j.jaci.2021.01.025

**Published:** 2021-06

**Authors:** Nandinee Patel, Daniel C. Adelman, Katherine Anagnostou, Joseph L. Baumert, W. Marty Blom, Dianne E. Campbell, R. Sharon Chinthrajah, E.N. Clare Mills, Bushra Javed, Natasha Purington, Benjamin C. Remington, Hugh A. Sampson, Alexander D. Smith, Ross A.R. Yarham, Paul J. Turner

**Affiliations:** a National Heart and Lung Institute, Imperial College London, London, United Kingdom; b Aimmune Therapeutics, Brisbane, Calif; c Section of Allergy and Immunology, Baylor College of Medicine, Houston, Tex; d Section of Allergy and Immunology, Department of Pediatrics, Texas Children's Hospital, Houston, Tex; e Food Allergy Research and Resource Program, University of Nebraska, Lincoln, Neb; f The Netherlands Organisation of Applied Scientific Research, Utrecht, The Netherlands; g Department of Allergy and Immunology, The Children’s Hospital at Westmead, Sydney, Australia; h DBV Technologies, Montrouge, France; i Sean N. Parker Center for Allergy and Asthma Research, Stanford, Calif; j Division of Pulmonary, Allergy, and Critical Care Medicine, Stanford University, Stanford, Calif; k Division of Infection, Immunity and Respiratory Medicine, Manchester Institute of Biotechnology, University of Manchester, Manchester, United Kingdom; l Department of Medicine, Quantitative Sciences Unit, Stanford University School of Medicine, Stanford, Calif; m Division of Pediatric Allergy and Immunology, Icahn School of Medicine at Mount Sinai, New York, NY; n Food Standards Agency, London, United Kingdom

**Keywords:** Eliciting dose, peanut allergy, thresholds, oral food challenge, precautionary allergen labeling, AIT, Allergen immunotherapy, DBPCFC, Double-blind placebo-controlled food challenge, ED, Eliciting dose, ED_01_, Amount of allergen expected to cause objective symptoms in 1% of the population with that allergy, ED_05_, Amount of allergen expected to cause objective symptoms in 5% of the population with that allergy, FC, Food challenge, IPD, Individual participant data, LOAEL, Lowest observed adverse effect level, PRACTALL, Practical Allergy, WAO, World Allergy Organization

## Abstract

**Background:**

Eliciting doses (EDs) (eg, ED_01_ or ED_05_ values, which are the amounts of allergen expected to cause objective symptoms in 1% and 5% of the population with an allergy, respectively) are increasingly being used to inform allergen labeling and clinical management. These values are generated from food challenge, but the frequency of anaphylaxis in response to these low levels of allergen exposure and their reproducibility are unknown.

**Objective:**

Our aim was to determine (1) the rate of anaphylaxis in response to low-level peanut exposure and (2) the reproducibility of reaction thresholds (and anaphylaxis) at food challenge.

**Methods:**

We conducted a systematic review and individual participant data meta-analysis of studies that reported at least 50 individuals with peanut allergy reacting to peanut at double-blind, placebo-controlled food challenge (DBPCFC) and were published between January 2010 and September 2020. Risk of bias was assessed by using National Institute for Clinical Excellence methodologic checklists.

**Results:**

A total of 19 studies were included (covering a total of 3151 participants, 534 of whom subsequently underwent further peanut challenge). At individual participant data meta-analysis, 4.5% (95% CI, 1.9% to 10.1%) of individuals reacted to 5 mg or less of peanut protein with anaphylaxis (moderate heterogeneity [*I*^*2*^ = 57%]). Intraindividual thresholds varied by up to 3 logs, although this variation was limited to a half-log change in 71.2% (95% CI, 56.2% to 82.6%) of individuals. In all, 2.4% (95% CI, 1.1% to 5.0%) of patients initially tolerated 5 mg of peanut protein but then reacted to this dose at subsequent challenge (low heterogeneity [*I*^*2*^ = 16%]); none developed anaphylaxis.

**Conclusion:**

Around 5% of individuals reacting to an ED_01_ or ED_05_ level of exposure to peanut might develop anaphylaxis in response to that dose. This equates to 1 and 6 anaphylaxis events per 2500 patients exposed to an ED_01_ or ED_05_ dose, respectively, in the broader population of individuals with peanut allergy.

## Graphical abstract

**Figure:**
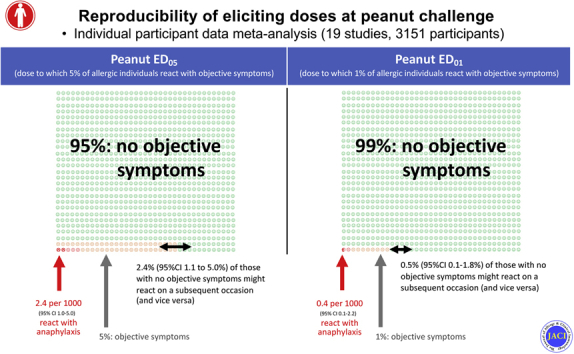

Food allergy is a global issue, affecting the international food supply, public health agencies, and government regulators. Historically, those with food allergies have been managed passively (ie, through dietary avoidance and provision of rescue medication such as self-injectable epinephrine), which is not a treatment strategy. However, our approach to food allergy is now rapidly shifting toward an approach in which active patient management (through interventions such as food allergy desensitization) and primary prevention are becoming commonplace. Some clinicians now advocate the use of food challenges (FCs) (including single-dose FCs) to establish a “safe” threshold for any given individual with a food allergy, which can then inform dietary allergen avoidance.^1^^,^^2^ In addition, allergen immunotherapy is now an established option to increase a patient’s reaction threshold and reduce the risk posed by accidental allergen exposure.

There is increasing interest in the application of scientific approaches to allergen risk assessment and management to improve allergen declarations for foods. Food businesses and some national regulators are increasingly embracing the use of “eliciting dose” (ED) data (derived from oral FC results) to inform allergen risk management in industry, such as the need for precautionary allergen labeling.^3^, ^4^, ^5^, ^6^, ^7^, ^8^ Although some gaps in knowledge remain, these approaches typically use the estimated elicited doses (EDs) at which 5% and 1% of the population with an allergy will experience objective symptoms (the ED_05_ and ED_01_, respectively).^3^, ^4^, ^5^ Although there is a consensus that zero risk is not realistic or achievable,^7^, ^8^, ^9^ the level of risk that is acceptable to consumers and regulators remains unclear.^8^^,^^9^ Although most consumers with an allergy may believe that mild, self-limiting symptoms in response to these low levels of allergen exposure are acceptable, others may not. Some consumers will still experience significant symptoms, and the proportion of these reactions that might constitute anaphylaxis is unclear. Furthermore, the stability or reproducibility of reaction thresholds (the minimum ED causing an objective allergic reaction) for individuals with an allergy is unknown: a consumer with an allergy may tolerate an ED_05_ exposure on 1 occasion but not on another. This may be due to the impact of “cofactors” (such as exercise, sleep deprivation, and intercurrent infection), or it may be independent of any cofactor.^10^ This is important, as the proportion of individuals with an allergy who react to an ED_05_ is unlikely to be static, as a result of which more than 5% of the population with an allergy might conceivably react to an ED_05_ level at some stage.

The past decade has seen a number of published phase 2 and phase 3 studies assessing the efficacy of allergen immunotherapy (AIT) for food allergy—to peanut in particular—which has considerably increased the available data relating to reaction thresholds in individuals with peanut allergy. In this analysis, we undertook a systematic review and meta-analysis of individual participant data (IPD)^11^ to inform these knowledge gaps by evaluating the proportion of reactions at low levels of allergen exposure that might be classified as anaphylaxis, as well as the reproducibility of individual reaction thresholds and the occurrence of anaphylaxis over time.

## Methods

We undertook a systematic review of the literature to identify studies that have undertaken double-blind, placebo-controlled FCs (DBPCFCs) in individuals with peanut allergy (adults and children) conducted in a manner consistent with international consensus criteria.^12^ Study sponsors and/or authors were contacted and asked to provide both aggregate and (in the case of individuals who underwent repeat peanut challenge) anonymized IPD that could then be included for meta-analysis. This review was undertaken and reported in accordance with the Preferred Reporting Items for Systematic Reviews and Meta-Analyses–for Individual Patient Data Statement.^11^

### Search strategy

We searched MEDLINE for articles that were published between January 2010 and September 2020 and described DBPCFC to peanut; we used the search terms *double-blind* and *peanut*. There was no registered protocol for this review, but the methods and analyses were planned *a priori*. No language restrictions were made, and we planned to include non–English language articles if they met our inclusion criteria. Abstracts were independently screened by 2 authors, and disagreements were resolved by discussion. We also reviewed the reference lists of the included studies and review articles to identify other relevant studies.

### Study selection

The inclusion criteria were as follows: (1) Participants: children and/or adults with suspected peanut allergy; (2) Intervention: DBPCFC to peanut undertaken for diagnostic purposes or to determine baseline reactivity before immunotherapy, in at least 50 subjects; (3) Outcomes: study-defined cumulative ED (either maximum tolerated dose or reaction threshold dose, consistent with Practical Allergy [PRACTALL] consensus criteria^12^) or lowest observed adverse effect level (LOAEL); occurrence of anaphylaxis. Studies needed to satisfy all 3 inclusion criteria to be included. The discrete ED_05_ for peanut is estimated to be 2.1 (95% CI, 1.2 to 4.6) mg of protein.^4^ To reduce the effect of left censoring, we therefore excluded any study in which the first challenge dose was more than 5 mg of peanut protein,.^3^, ^4^, ^5^ When more than 1 report included the same individuals with an overlapping study period, we included the data from the report with the largest number of individuals in which we could be certain that no duplication was present.

### Data extraction and analyses

Study sponsors and/or authors were contacted to confirm the data extracted and, where indicated, review the individual-level data under confidentiality agreements. Analyses were planned prospectively. The previously published estimates for discrete ED_01_ and ED_05_ for peanut are 0.2 (95% CI, 0.1 to 0.4) and 2.1 (95% CI, 1.2 to 4.6) mg of protein, respectively.^4^ For each study, we therefore extracted (1) the number of participants experiencing objective symptoms and/or meeting the study-defined challenge stopping criteria to a discrete dose of 1 mg or less and 5 mg or less of peanut protein and (2) the proportion of those with anaphylaxis (as defined by the authors for each included study). The different definitions used for anaphylaxis are reported in Table I.^4^^,^^5^^,^^13^, ^14^, ^15^, ^16^, ^17^, ^18^, ^19^, ^20^, ^21^, ^22^, ^23^, ^24^, ^25^, ^26^, ^27^, ^28^, ^29^, ^30^, ^31^, ^32^, ^33^ Where individual patient symptom data were available, anaphylaxis was determined by 2 independent investigators (P.J.T. and N.P.) according to the World Allergy Organization (WAO) 2020 consensus criteria.^20^ Data were extracted in duplicate: we extracted outcome data that adhered to the intention-to-treat principle in preference to data based on per-protocol analyses. Any discrepancies identified between the extracted data and published data were resolved by discussion and/or by contacting authors for clarifications. Risk of bias was assessed by using the National Institute for Clinical Excellence methodologic checklists for cohort studies.^34^ Rates were pooled across studies by using a generalized linear mixed model in R software (metaprop function, metafor package, and logit transformation with a random intercept logistic regression model for the summary estimate, with a continuity correction of 0.5). This approach avoids many of the issues surrounding use of transformations when undertaking meta-analyses of proportions.^35^^,^^36^ Heterogeneity was quantified by using the *I*^*2*^ statistic. We conducted meta-analysis even if significant heterogeneity was seen between study estimates, as is the norm when conducting meta-analysis of proportions. The statistical program used for meta-analysis was R, version 4.0.3 (R Project). Binomial confidence intervals were calculated by using the Clopper-Pearson interval. Statistical significance was set at a 2-sided *P* value less than .05. Sensitivity analyses were performed to (1) assess for any difference between those studies reporting LOAELs (defined according to Westerhout et al^37^) and those that used study-defined dose-limiting symptoms and (2) assess for any impact of the different anaphylaxis criteria used by individual studies on the overall pooled estimate.

**Table I:** Characteristics of included cohorts

Study	n		Age of cohort	Inclusion criteria	DBPCFC protocol (mg of peanut protein)	Threshold definition	Anaphylaxis definition used	Median cumulative dose	Number with symptoms in response to a ≤5-mg discrete dose	
	Published	Data available							Objective symptoms, no. (%)	Study-defined anaphylaxis
										Symptoms
Taylor et al, 2010^13^	286	283	Range, 1-48 y; median, 7 y	Routine diagnostic FC	Various, 0.025-2.5 as initial dose; 15-min intervals	LOAEL	CVS/lower respiratory	125 mg (IQR = 16-241)	22 (8%)	1 case of asthma with dyspnea
Blom et al, 2013^14^	135	123	Range, 2-18 y; median, 7 y	Routine diagnostic FC	1.7, 3.5, 14, 70, 139, and 351; 30-min intervals	LOAEL	CVS/slower respiratory	144 mg	8 (7%)	None
Van Erp et al, 2013^15^	109	109	Median, 7 y (IQR, 5-9 y)	Routine diagnostic FC	0.005, 0.05, 0.25, 0.5, 5, 50, 150, 500, and 1500; 15- to 30- min intervals	LOAEL	Sampson^16^ grade 4/5	706 mg (IQR = 206-2206)	8 (7%)	2 cases: 1 with LE/wheeze and 1 with LE/wheeze
STOP-II 2014^17^	99	99	Range, 7-16 y; median 12 y	Reaction to ≤1455 mg	5, 50, 100, 300, and 1000; 20- to 30-min intervals	DLS	NIAID^18^	55 mg (IQR = 5-1400)	12 (12%)	None
EuroPrevall 2015^4^^,^^5^	51	43	Median 8 y (IQR = 2-31 y)	Diagnostic FC	0.003, 0.03, 0.3, 3, 30, 100, 300, 1000, and 3000; 20-min intervals	LOAEL	CVS/lower respiratory	1433 mg	3 (6%)	None
Klemans et al, 2015^19^	100	100	Range, 16-64 y; median, 24 y	Routine diagnostic FC	0.005, 0.05, 0.25, 0.5, 5, 50, 150, 500, and 1500; 15- to 30-min intervals (26% received 0.03, 0.1, 0. 3, 1, 3, 10, 30, 100, 300, and 1000 mg)	LOAEL	Consistent with WAO^20^^,^∗	206 mg	6 (6%)	1 case with OAS, LE, and AP
Kukkonen et al, 2015^21^	69	69	Range, 6-18 y; median, 8 y	Reaction to ≤1255 mg	5, 50, 200, and 1000; 30-min intervals	DLS	Hourihane^22^	55 mg	9 (13%)	1 case with LE/mild wheeze.
FAHF-2 2015^23^	50	50	Range, 12-45 y; median 16 y	Reaction to ≤2000 mg	1, 5, 15, 50, 75, 100, 250, 500, and 1000; 10- to 15-min intervals	DLS	NIAID^18^	146 mg	13 (26%)	3 cases; all lower respiratory + gut
ARC001 2017^24^	55	55	Range, 4-26 y; median 8 y	Reaction to ≤143 mg	3, 10, 30,and 100; 20-to 30-min intervals	DLS	NIAID^18^	43 mg (range, 13-143)	0	None
VIPES 2017^25^	221	221	Range, 6-55 y; median 11 y 84% <18 y	Reaction to ≤444 mg	1, 3, 10, 30, 100, and 300; 30-min intervals	DLS	Consistent with WAO^20^^,^∗	144 mg (IQR = 44-444)	20 (9%)	3 cases: 1 with OAS, repetitive vomiting; 1 with nausea, AP, wheeze, and vomiting; and 1 with OAS, LE, AP, and nausea
TAKE-AWAY 2017^26^	96	96	Range, 5-15 y; median 9 y	Reaction to ≤144 mg	3, 10, 30, 100, 300, 1000, and 3000; 30- to 60-min intervals	DLS	CVS/lower respiratory	44 mg (IQR = 4-144)	19 (20%)	1 case with wheeze
Purington et al, 2018^27^	347	307	Range, 1-52 y; median 9 y	Routine diagnostic FC	0.1, 1.7, 5, 20, 50, 100, 100, 100, 123; 15-min intervals	DLS	NIAID^18^	75 mg	57 (16%)	None
PALISADE 2018^28^	551	551	Range, 4-55 y; 90% < 18 y	Reaction to ≤144 mg	1, 3, 10, 30, and 100; 20- to 60-min intervals	DLS	NIAID^18^ and SAE definition	44 mg (IQR = 4-144)	66 (12%)	None
PEPITES 2019^29^	356	356	Range, 4-11 y; median, 7 y	Reaction to ≤444 mg	1, 3, 10, 30, 100, and 300; 30-min intervals	DLS	Consistent with WAO^20^^,^∗	144 mg (IQR = 44-444)	23 (6.5%)	4 cases: 1 with U/A, rhinitis, LE, and AP; 1 with LE, vomit, diarrhea, and OAS; 1 with U/A, rhinitis, and wheeze; and 1 with pruritus, rhinitis, and wheeze
TRACE 2019^30^	123	123	Range, 18-45 y; mean, 25 y	Reaction to ≤1433 mg	0.003, 0.03, 0.3, 3, 30, 100, 300, and 1000; 30- to 60-min intervals	DLS	Consistent with WAO^20^^,^∗	133 mg (IQR = 133-433)	4 (3%)	1 case of throat tightness, AP, rhinitis, vomit × 1, vocal hoarseness
BOPI 2019^31^	64	64	Range, 8-16 y; median 13 y	Reaction to ≤4443 mg	3, 10, 30, 100, 300, 1000, and 3000; 30- to 60-min intervals	DLS	Consistent with WAO^20^^,^∗	143 mg (IQR = 43-443)	4 (5%)	None
POISED 2019^32^	120	120	Range 7-55 y; median, 11 y; 69% <18 y	Reaction to ≤500 mg	5, 20, 50, 100, 100, and 100; 15- to 60-min intervals	DLS	Consistent with WAO^13^^,^∗	75 mg (IQR = 25-175)	12 (10%)	None
ARTEMIS 2020^33^	175	175	Range, 4-17 y; mean, 9 y	Reaction to ≤444 mg	1, 3, 10, 30, 100, and 300; 20- to 30-min intervals	DLS	NIAID^18^	44 mg (IQR = 14-44)	38 (22%)	1 case with OAS, wheeze, hypotension
UMCG 2020^4^	144	144	Range, 1-18 y; median, 8 y	Routine diagnostic FC	Before 2007: 1.75, 3.5, 14,70, 130, 350, and 570 mg After 2007: 0.6, 3, 10, 30, 100, 300, and 1000 mg	LOAEL	CVS/lower respiratory	95.8 mg	12 (8%)	None

All doses are expressed in milligrams of peanut protein.

*AP*, Abdominal pain; *CVS*, cardiovascular symptoms; *DLS*, dose-limiting symptoms; *IQR*, interquartile range; *LE*, symptom of laryngeal edema; *NIAID*, National Institute of Allergy and Infectious Diseases; *OAS*, oral allergy symptoms; *SAE*, severe adverse event; *U/A* urticaria/angioedema.

∗ Individual participant symptom data were available in these studies and used to reassign the occurrence of anaphylaxis (or not) according to the WAO 2020 criteria.

To assess the reproducibility of challenge thresholds over time within individuals, we extracted the IPD of individuals who underwent further FC following initial DBPCFC (conducted according to the same protocol) from relevant interventional studies (eg, participants who were randomized to a placebo control arm in studies of food allergy desensitization). The log fold change in reaction threshold for each subject was calculated. Normality of distribution was assessed by using the D'Agostino-Pearson test, after which the distributions were used in IPD meta-analysis. We included a sensitivity analysis to assess the degree of stability of reaction threshold in individuals with peanut allergy reacting to lower EDs (≤5 mg of peanut protein). Separately, we evaluated the reproducibility of the occurrence of anaphylaxis after a repeat exposure.

### Ethical approval

Ethical approval was not required, as this was a *post hoc* analysis of anonymized participant data from multiple clinical trials, each of which had its own individual ethics approval.

## Results

**Fig 1:**
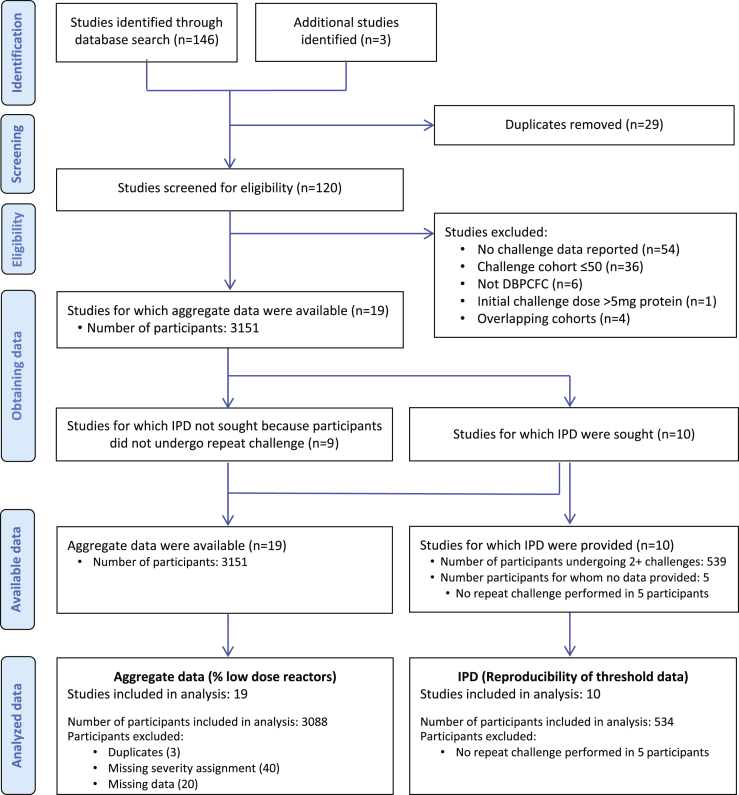
Preferred Reporting Items for Systematic Reviews and Meta-Analyses flow diagram.

A total of 19 studies incorporating a total of 3151 participants who underwent DBPCFC to peanut were identified as eligible for inclusion (Fig 1). The details of the individual studies appear in Table I, and the details risk of bias assessment are presented in Table E1. No study had a high risk of bias or poor external validity. In total, data were available for 3088 participants across 19 studies,^4^^,^^5^^,^^13^, ^14^, ^15^^,^^17^^,^^19^^,^^21^^,^^23^, ^24^, ^25^, ^26^, ^27^, ^28^, ^29^ and they formed the primary analysis cohort. Of these 3088 participants, 534 underwent subsequent repeat challenge; IPD were available for all 534 individuals.

### Anaphylaxis at low levels of allergen exposure

**Fig 2:**
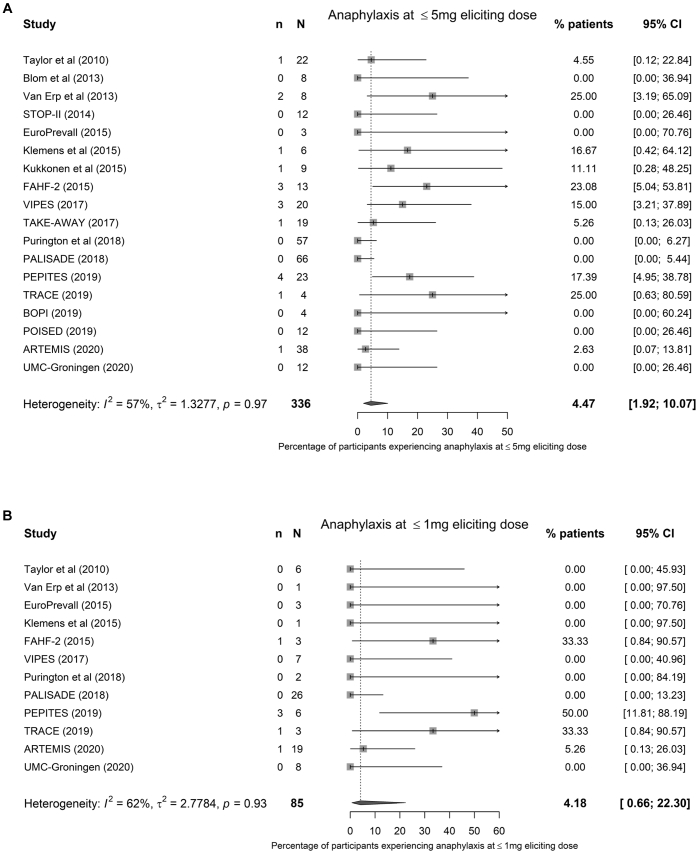
Meta-analysis of aggregate data from 19 studies assessing the proportion of individuals with peanut allergy reacting with objective symptoms in response to 5 mg or less (**A**) and 1 mg or less (**B**) of peanut protein who developed anaphylaxis at that dose.

**Fig E1:**
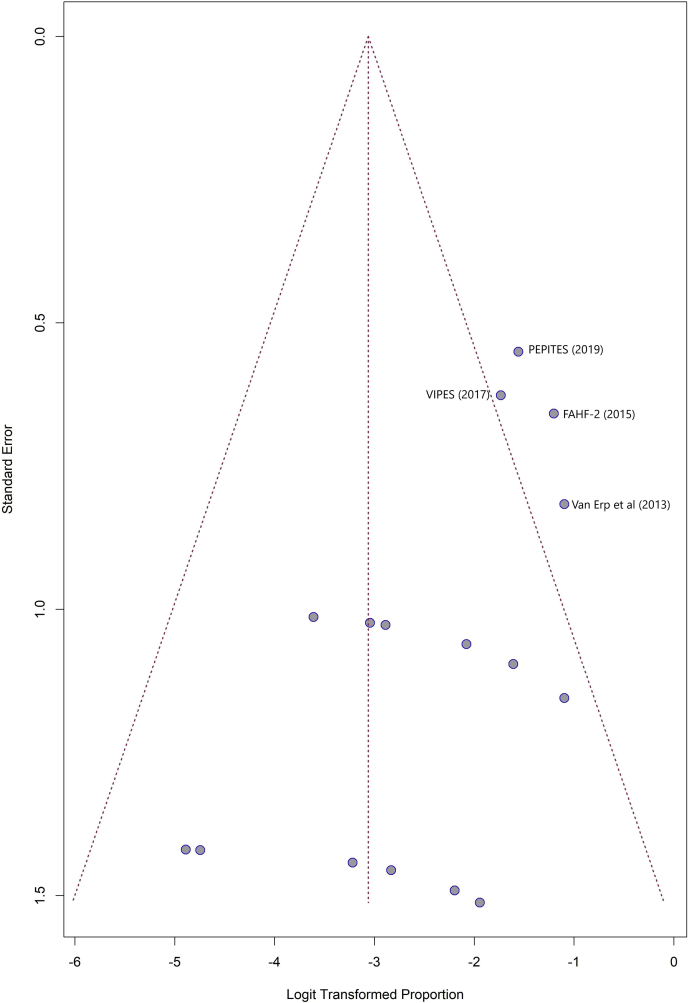
Funnel plot of the included studies. Note, however, that the presence of funnel plot asymmetry in meta-analyses of proportions does not necessarily indicate publication bias (Hunter et al, J Clin Epidemiol. 2014;67:897-903). In this case, there was no evidence of publication bias in terms of the rate of anaphylaxis in low-dose reactors, by size of study.

**Fig E2:**
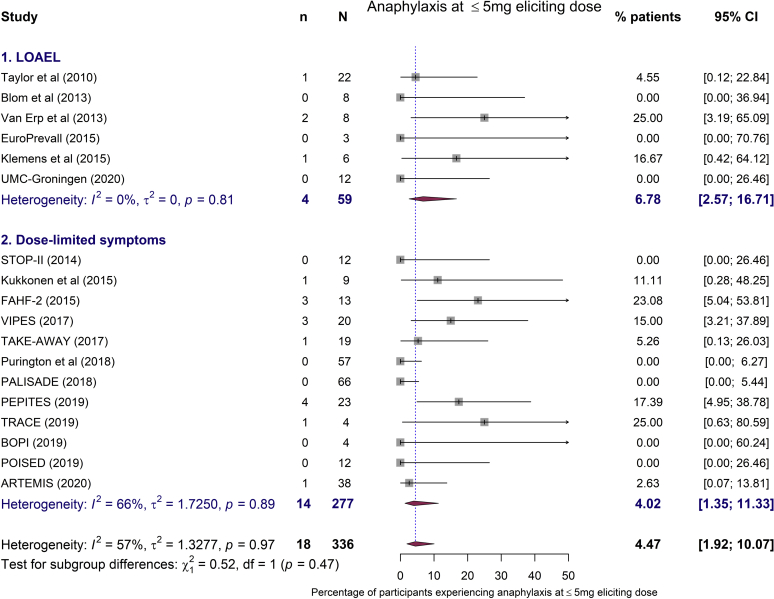
Meta-analysis of aggregate data from 19 studies assessing the proportion of individuals with peanut allergy reacting to 5 mg or less with anaphylaxis, divided by the criteria used to define threshold (1) LOAEL and (2) study-defined dose-limiting symptoms.

**Fig E3:**
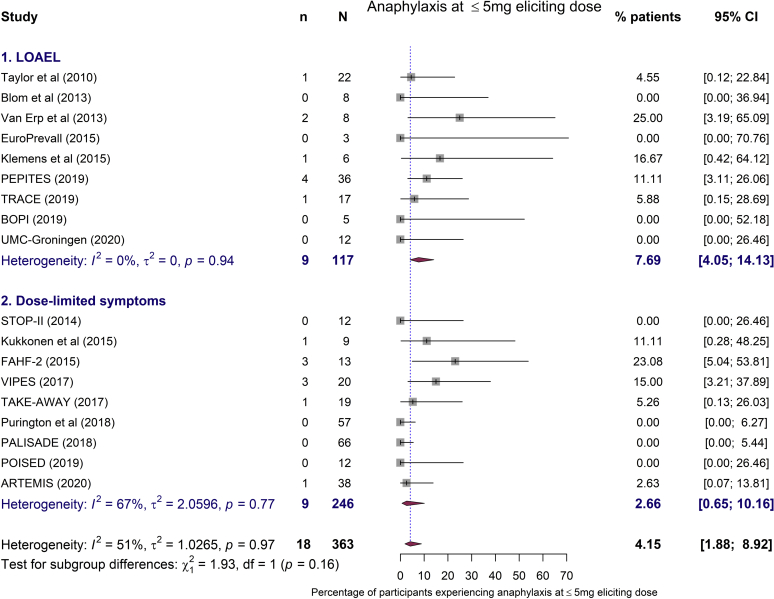
Sensitivity analysis of aggregate data from all 19 studies assessing the proportion of individuals with peanut allergy reacting to 5 mg or less of peanut protein with anaphylaxis, incorporating reanalyzed data from 3 studies (PEPITES, TRACE, and BOPI) by using LOAEL rather than dose-limiting symptoms to define reaction threshold.

Aggregate data were available from all 19 studies (Table I). Overall, 336 participants across 19 studies reacted (according to individual study-defined criteria) to 5 mg or less of peanut protein (see the funnel plot shown in Fig E1 [available in this article's Online Repository at www.jacionline.org]). At meta-analysis, 4.5% (95% CI, 1.9% to 10.1%) of individuals reacting to exposure to 5 mg or less of peanut protein (discrete dose) would be expected to develop anaphylaxis (moderate heterogeneity [*I*^*2*^ = 57%] (Fig 2, *A*). We did not identify any significant differences in estimates when comparing studies that used LOAEL with those using dose-limiting symptoms in a sensitivity analysis (see Fig E2 in this article's Online Repository at www.jacionline.org), although the meta-analysis suggested that the overall heterogeneity was due to interstudy differences in defining dose-limiting symptoms, as there was minimal heterogeneity when LOAEL criteria were used. In a further sensitivity analysis, IPD from 3 studies were reanalyzed to determine reaction thresholds based on published LOAEL criteria.^37^ This suggested that although some individual study estimates might change, overall, there was little change in the estimate of participants reacting to 5 mg or less of peanut protein with anaphylaxis, with a revised estimate of 4.2% (95% CI, 1.9% to 8.9%; *I*^*2*^ = 51%) (see Fig E3 in this article's Online Repository at www.jacionline.org).

In all, 12 studies included an initial challenge dose of 1 mg or less of peanut protein; at meta-analysis, 4.2% (95% CI, 0.7% to 22.3%) of reactions to 1 mg or less of peanut protein would be anaphylaxis (moderate heterogeneity [*I*^*2*^ = 56%]) (Fig 2, *B*). Because of fewer reactions at this level of allergen exposure, no sensitivity analyses were undertaken.

**Fig E4:**
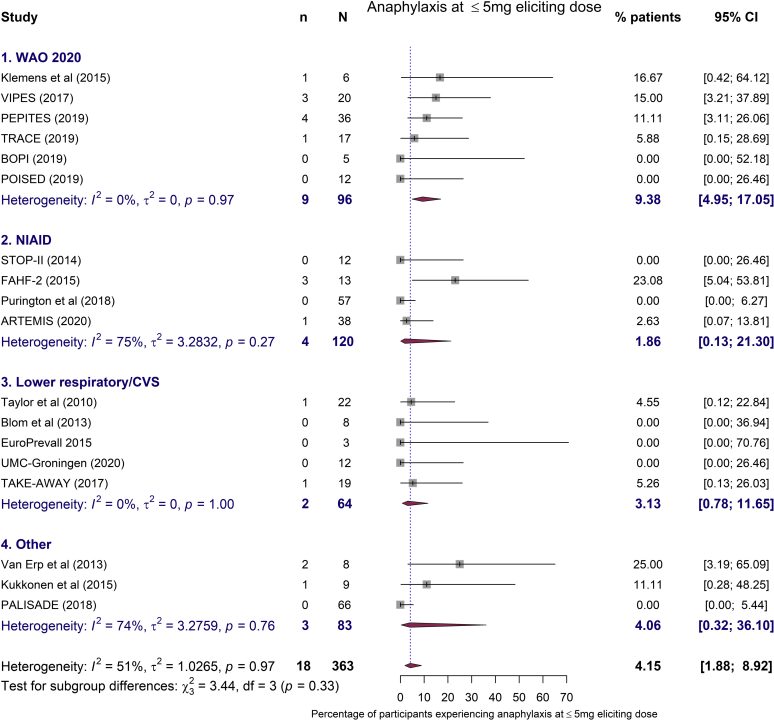
Sensitivity analysis of aggregate data from all 19 studies assessing the impact of different definitions of anaphylaxis on the pooled estimate for the proportion of individuals with peanut allergy reacting to 5 mg or less of peanut protein with anaphylaxis.

We undertook a sensitivity analysis to assess for any impact of the different definitions of anaphylaxis used by the individual studies (see Fig E4 in this article's Online Repository at www.jacionline.org). There was substantial heterogeneity in those studies using the National Institute of Allergy and Infectious Diseases definition for anaphylaxis,^18^ but overall, this did not affect the pooled estimate (*P* =.33; chi-square test). Furthermore, in a pooled analysis of the data available for IPD meta-analysis, 120 of 534 individuals reacted to a discrete dose of 5 mg or less of peanut protein; 7 developed anaphylaxis (as defined by the WAO 2020 criteria^20^), giving a rate of 5.8% (95% CI, 2.4% to 11.7%).

### Reproducibility of reaction thresholds

In all, 10 interventional studies included participants who underwent repeat FC; the interval between challenges varied, both within and between studies as reported in Table E1. Of these studies, 9 were clinical trials of food allergy desensitization, from which participants randomized to placebo treatment were included in the IPD meta-analysis. The tenth was the TRACE peanut study,^30^ in which adults with a positive DBPCFC to peanut were randomized to undergo repeat peanut challenge with or without cofactors (exercise, sleep deprivation). For the purpose of this analysis, we used data from the baseline DBPCFC and nonintervention challenge (without a cofactor), which for the majority of participants, was an open FC otherwise conducted according to an identical protocol as baseline DBPCFC with the same challenge-stopping criteria.

**Fig 3:**
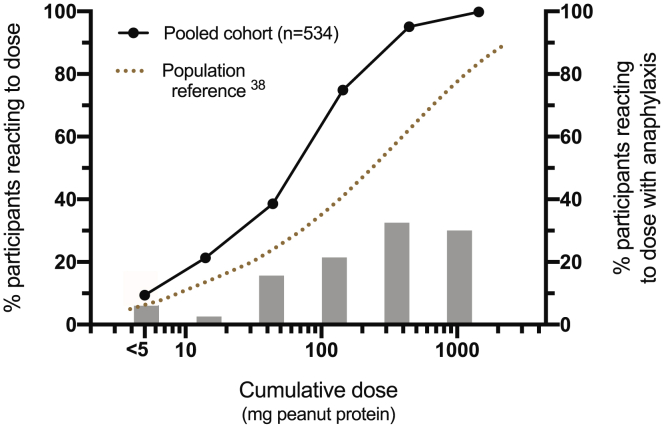
Dose distribution for reaction threshold at baseline DBPCFC in 534 participants included in the IPD meta-analysis (a pooled cohort of 10 studies) who underwent 2 challenges. The proportion of participants reacting with anaphylaxis (defined according to the WAO 2020 criteria^13^) at each dosing level is also shown. Population reference distribution derived from Houben et al.^38^

The dose distributions for baseline DBPCFC in participants in the pooled cohort are shown in Fig 3 together with the proportion of participants reacting at each dosing level with anaphylaxis (defined according to WAO 2020 criteria^20^). The median cumulative reaction threshold for the combined cohort was 143 mg of peanut protein (interquartile range, 27 to 144 mg), whereas the overall rate of anaphylaxis was 19.6%. Compared with the published dose distributions for individuals with peanut allergy,^38^ there was evidence of skewing toward a more sensitive population, which is not unexpected given the inclusion criteria of the included studies.

**Fig 4:**
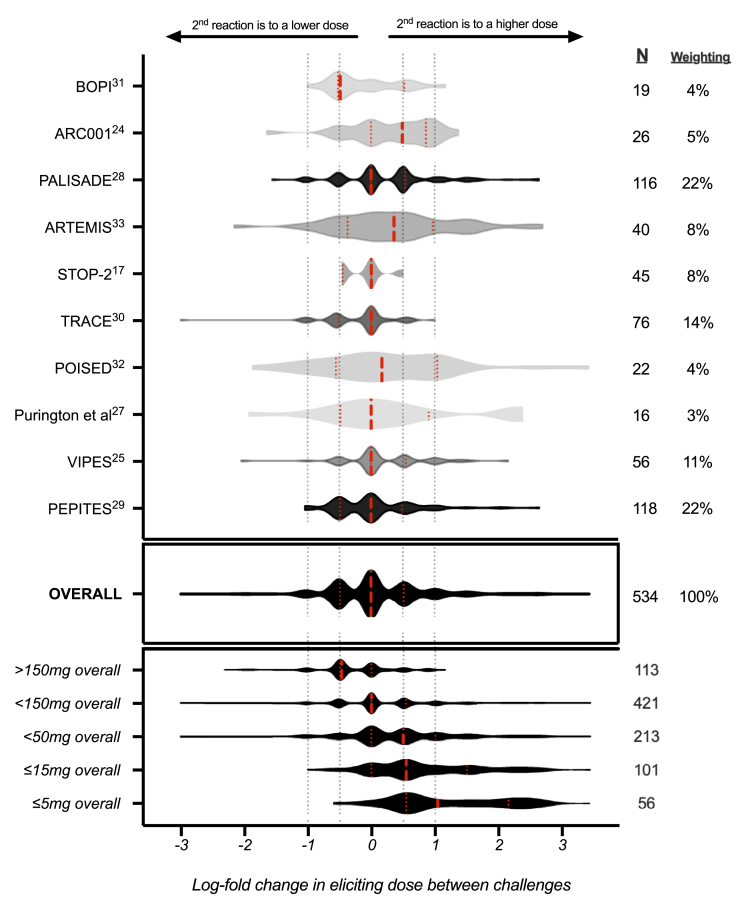
Violin plot of the distributions of log change in reaction thresholds (from initial DBPCFC to repeat FC) for study participants within each included cohort. A half-log change in ED is equivalent to a shift in reaction threshold by 2 dosing increments when a PRACTALL-based semilog regimen is used. The red dashed line represents the median, and the red dotted line represents the interquartile range.

The distributions of log change in reaction thresholds for study participants within each included cohort are shown in Fig 4. These distributions were normally distributed (according to the D'Agostino-Pearson test) for all cohorts apart from the TRACE cohort (skewing toward a decrease in reaction thresholds with repeat challenge) and PALISADE and PEPITES cohorts (skewing toward an increase in reaction threshold). These distributions were then analyzed by IPD meta-analysis to determine the proportion of participants with a change in reaction threshold at repeat challenge and whether this proportion differed in patients who reacted to lower levels of peanut exposure (see Table II).

**Table II:** Proportions of participants with a change (or no change) in threshold, (overall and cohorted into those with lower reaction thresholds to peanut), by IPD meta-analysis results

Cumulative reaction threshold at initial challenge (mg of peanut protein)	At IPD meta-analysis, the proportion of participants (and 95% CI) with						
	Increase in threshold		**No change in threshold**	Decrease in threshold		**±Max half-log change**	± Max 1-log change
	> Half-log	Any		Any	> Half-log		
Any _(n = 534)_	18.3% (11.0-28.9)	35.5% (26.1-46.3)	***32.2% (25.3-40.0)***	30.3% (24.5-36.9)	8.2% (4.5-14.5)	***71.2% (56.2-82.6)***	91.2% (84.1-95.3)
>150 mg (ie, no objective symptoms in response to ½ peanut) _(n = 113)_	3.2% (0.5-16.5)	15.6% (10.3-23.9)	***28.3% (20.8-37.3)***	59.6% (42.1-75.0)	11.9% (6.2-21.6)	***85.0% (77.1-90.4)***	96.5% (90.4-98.8)
<150 mg _(n = 423)_	22.5% (14.5-33.2)	40.1% (30.7-50.2)	***34.5% (25.6-44.7)***	23.0% (19.3-27.3)	7.0% (3.5-13.6)	***68.1% (53.9-79.7)***	89.9% (82.9-94.2)
<50 mg _(n = 213)_	35.7% (28.1-44.1)	56.6% (47.0-65.8)	***28.7% (21.8-36.7)***	15.0% (10.8-20.5)	6.6% (3.9-10.9)	***56.9% (47.7-65.6)***	81.7% (75.9-86.3)
≤15 mg _(n = 101)_	50% (38-62)	72% (63-80)	***21% (12-35)***	5.0% (2.1-11)	2.8% (0.7-11)	***44% (30-59)***	68% (59-77)
≤5 mg _(n = 56)_	63% (47-77)	82% (70-90)	***10% (2.6-31)***	5.4% (1.7-15)	5.4% (1.7-15)	***24% (8-53)***	61% (47-73)

*max*, Maximum.

A half-log or 1-log change in threshold is equivalent to a shift in reaction threshold by 1 or 2 dosing increments when a challenge protocol based on PRACTALL is used. Boldface highlights "no change in threshold" and "+/- max 1/2-log change" which are arguably the most important outcome measures.

**Fig E5:**
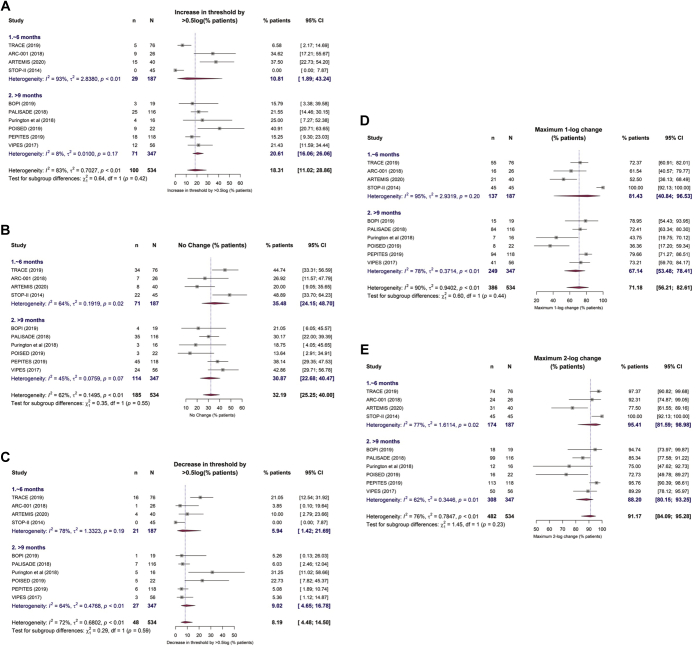
Sensitivity analysis of individual participant data from 10 studies assessing the reproducibility of challenge threshold, by time interval in between the 2 FC occasions (approximately 6 months [range 3-9] versus more than 9 months. **A**, Increase in challenge threshold by more than a half-log change. **B**, No change in threshold; **C**, Decease in challenge threshold by more than a half-log. **D**, Maximum change log in challenge threshold by plus or minus 1. **E**, Maximum change in challenge threshold by plus or minus 2 logs.

Overall, 71.2% (95% CI, 56.2% to 82.6%) of participants reacted at repeat challenge to the same dose plus or minus a half-log versus at initial challenge, which is equivalent to 1 dosing interval with use of a PRACTALL-based semilog dosing regimen (eg, a change in threshold from 100 mg to 300 mg of peanut protein). Analyzing more sensitive individuals who reacted to lower doses, we found that as the ED became less, the reproducibility of the triggering dose decreased: individuals reacting to 5 mg or less of peanut protein with objective symptoms were more likely to react to higher doses at repeat challenge with a greater than a half-log increase. We undertook a sensitivity analysis to assess whether there was any evidence for an impact of the interval between the 2 challenge occasions on the reproducibility of challenge threshold. We did not identify a statistically significant difference between the pooled estimates from those studies in which the dosing interval was approximately 6 months (range 3-9 months) as opposed to more than 9 months (*P* > .05 [see Fig E5 in this article's Online Repository at www.jacionline.org]).

**Fig E6:**
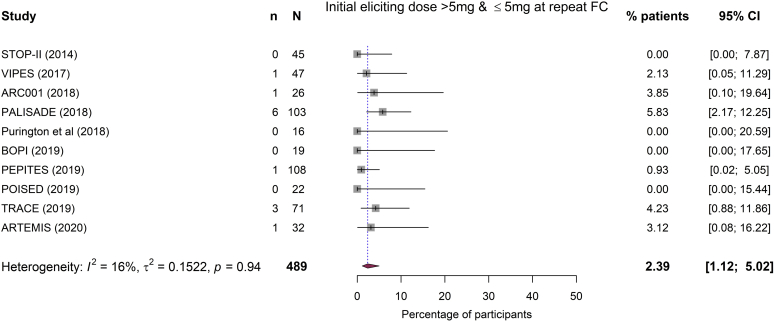
Meta-analysis of individual participant data from 10 studies assessing the proportion of individuals with peanut allergy who reacted to more than 5 mg of peanut protein at initial challenge but then reacted to 5 mg or less at the subsequent challenge.

In terms of protecting the consumer with a food allergy from low-dose exposures, 1 concern is that an individual who, for example, tolerates an ED_05_ level of exposure on 1 occasion might react to a lower amount on another. To address this, we undertook an IPD meta-analysis to assess the proportion of participants who reacted to more than 5 mg of peanut protein at initial challenge but then reacted to 5 mg or less at the subsequent challenge. At meta-analysis, 2.4% (95% CI, 1.1% to 5.0%) of individuals with peanut allergy reacted to peanut protein in a dose of 5 mg or less at subsequent challenge, having initially tolerated this dose (low heterogeneity [*I*^*2*^ = 16%]) (see Fig E6 in this article's Online Repository at www.jacionline.org); none developed anaphylaxis. Only 3 participants in the combined data set had a reaction to 1 mg or less of peanut protein after having tolerated this dose initially, which is equivalent to a rate of 0.5% (95% CI, 0.1% to 1.8%); no meta-analysis was performed owing to the small numbers involved.

### Recurrence of anaphylaxis

**Fig 5:**
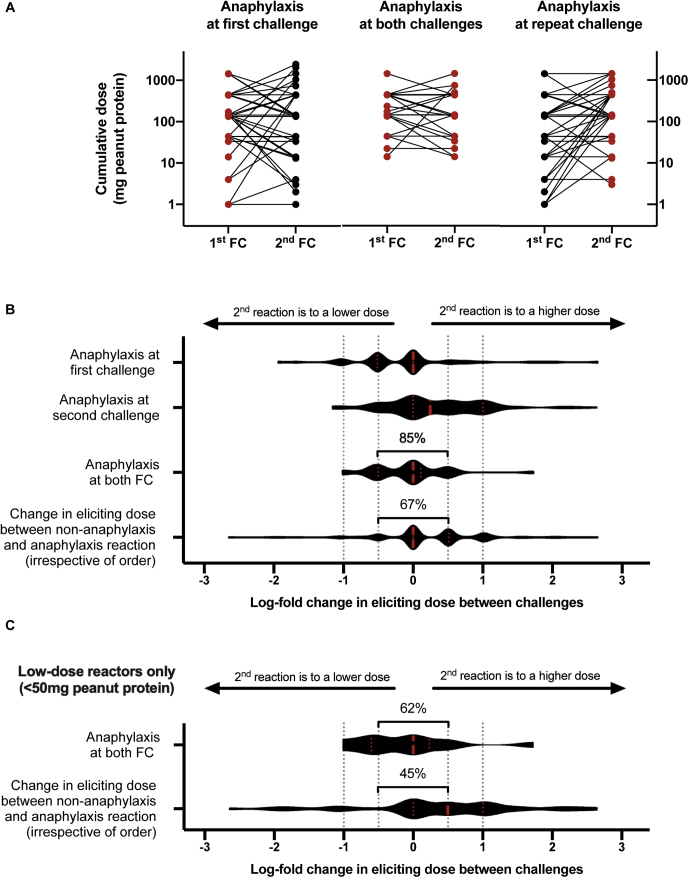
Change in reaction threshold in those study participants who underwent 2 peanut challenges and experienced anaphylaxis on at least 2 occasions. **A**, Absolute change in threshold. **B**, Violin plot of the distributions of log-fold change in reaction thresholds between first and second challenge, unless otherwise stated. **C**, Violin plot of the same outcomes in those individuals with a cumulative reaction dose of peanut protein lower than 50 mg. Red dashed line represents the median, and red dotted lines represent the interquartile range.

Lastly, we analyzed data from participants who underwent 2 challenges and developed anaphylaxis (according to WAO 2020 consensus criteria^20^) on at least 1 occasion. Data were available from 8 studies, yielding a total of 152 of 467 participants (33%) who had at least 1 anaphylaxis reaction (Table II). For the pooled analysis, the change in ED is shown in Fig 5, *A*-*C*. Just as there was variability in the reproducibility of the ED (causing any objective symptoms), we also found similar variability in the reproducibility of the dose at which participants experienced anaphylaxis: 33 participants developed anaphylaxis at both FCs, of whom 28 participants (85% [95% CI 68% to 95%]) had their second anaphylaxis reaction at a threshold that was equivalent to that of the index reaction, plus/minus a half-log difference. Importantly, 75% (95% CI, 65% to 83%) of those with anaphylaxis at the initial FC did not develop anaphylaxis in response to the same (or a lower) dose at subsequent exposure. Similarly, 26 of 119 participants (22% [95% CI, 15% to 30%]) developed anaphylaxis in response to a dose of peanut less than that which caused a nonanaphylaxis reaction on another occasion. The risk of anaphylaxis in response to a lower dose at second challenge in low-dose reactors (individuals reacting at first challenge to less than 50 mg of peanut protein) was significantly lower than the risk in the overall cohort (*P* < .05; Fisher exact test [Table III]) but similar for anaphylaxis in response to the same level of peanut exposure.

**Table III:** Probability of the occurrence of anaphylaxis at a subsequent FC event

Symptoms at subsequent FC	Anaphylaxis			Non-anaphylaxis in response to the same or a higher dose
Symptoms at at index FC	In response to a lower dose (compared with the response to the index reaction)	In response to a lower or same level of exposure	In response to a higher dose∗	
Anaphylaxis				
In response to any dose (n = 100)	12% (6-20)	25% (17-35)	8% (4-15)	35% (26-45)
In response to <50 mg (n = 23)	4.4% (0.1-22)	22% (7-44)	9% (1-28)	43% (23-66)
Nonanaphylaxis				
In response to any dose (n = 52)	19% (6-20)	50% (36-64)		
In response to <50 mg (n = 23)	4.4% (0.1-22)	30% (13-53)		

In all data cells, the intervals in parentheses are 95% CIs.

∗ These data must be interpreted with caution, as the risk of anaphylaxis in response to higher doses would have reduced by the challenge being terminated in many individuals at the onset of objective symptoms (before the onset of anaphylaxis), thus potentially limiting reaction severity.

## Discussion

The use of reference doses (based on the ED_01_ and ED_05_ values generated from FC data) to inform allergen risk management is increasing; however, like the use of precautionary allergen labeling, this area is currently unregulated in most countries, in part because of knowledge gaps, which include the risk of anaphylaxis in response to low-level allergen exposure.^8^^,^^39^ In this IPD meta-analysis of threshold and symptom data from more than 3000 DBPCFCs to peanut, we found that approximately 4% to 5% of individuals with an allergy who react to ED_01_ or ED_05_ levels of peanut with objective symptoms will experience anaphylaxis. Within the overall population of individuals with peanut allergy, this equates to an ED_05_ level of peanut exposure causing anaphylaxis in 2.4 individuals per 1000 with allergy (95% CI = 1.0-5.0) and an ED_01_ exposure causing anaphylaxis in 0.4 individuals per 1000 (95% = CI 0.1-2.2).

Establishing the reproducibility of the FC procedure to determine reaction thresholds in patients with a food allergy is a prerequisite for the optimal use of threshold data in allergen risk management. Our data show that around 70% of individuals with peanut allergy have a degree of “shift” of up to a half-log in clinical reactivity (equivalent to 1 dosing increment when using a semilog-based dosing regimen, such as that recommended by the PRACTALL consensus^12^), with approximately 20% reacting with up to a 10-fold shift and 10% demonstrating a greater change. In addition, 2.4% (95% CI = 1.1% to 5.0%) of individuals reacting to an ED_05_ level of exposure on 1 occasion might have previously tolerated this dose (and *vice versa*, so that at any 1 time only 5% of individuals with peanut allergy would react to an ED_05_ level of exposure); however, in our combined cohort, no one developed anaphylaxis. We found a similar variability in the occurrence of anaphylaxis, with 29% (95% CI = 21%-38%) of individuals reacting to level of peanut exposure with anaphylaxis on 1 challenge occasion, but not on another. These data are summarized in the graphical abstract.

### Allergen risk management

These data are crucial in developing an evidence-based approach to allergen risk management in food production. Currently, the use of risk-mitigating strategies (such as measures to reduce allergen cross-contamination on shared production lines and use of precautionary allergen labeling and food recalls) may not be evidence based. A number of initiatives, including the Voluntary Incidental Trace Allergen Labeling scheme^3^^,^^4^ and probabilistic risk assessment models (such as that proposed by the Integrated Approaches to Food Allergen and Allergy Risk Management collaboration),^40^^,^^41^ provide evidence-based risk assessment approaches for allergen risk management. These models need to consider not only the risk of a clinical reaction but also the severity of those symptoms.^10^

Our data indicate that the use of ED_05_ and ED_01_ levels to inform such approaches is justified. At an ED_05_ level of exposure, 5% of individuals with an allergy would still have a reaction with objective symptoms, and around 5% of these reactions would be anaphylaxis, which is equivalent to 6 anaphylaxis reactions per 2500 individuals exposed to an ED_05_ level. At an ED_01_ level, the expected rate of anaphylaxis would be 1 per 2500. On the basis of the reaction symptoms reported in this analysis, the vast majority would be at the less severe end of the anaphylaxis spectrum (eg, responsive to a single dose of epinephrine). There is a need to determine which of these levels of risk may be acceptable to patients, given the potential significant benefits of increased food choice and consumer confidence that allergen labeling is based on a proper risk assessment procedure.^8^^,^^41^ Furthermore, our data show that such reactions may be limited to those individuals who can be readily identified as “very low-dose reactors” through single-dose challenges to an ED_05_ dose.^1^ Although up to 5% of patients with peanut allergy who were included in this analysis reacted to ED_05_ or lower levels at a subsequent challenge after having tolerated this level of allergen exposure in the first instance, none developed anaphylaxis. Identifying patients who are unable to tolerate ED_05_ levels of allergen exposure may therefore facilitate targeted dietary advice for these patients to maintain strict allergen avoidance,^1^ whereas allowing the majority to adopt a greater level of dietary freedom and, most importantly, providing reassurance as to the very low risk of a more significant reaction due to accidental exposure when consuming food products that have been subjected to an evidence-based risk management process.

These data also demonstrate that anaphylaxis occurs at all levels of allergen exposure. This is an important observation, as it has been suggested that patients who react to lower doses are more likely to experience severe reactions. In a cohort of 117 preschool-age children with peanut allergy, Santos et al observed a relationship between clinical severity at FC and cumulative threshold dose^42^; however, the study utilized a dosing regimen (a starting dose of 33 or 100 mg) that would have resulted in significant left censoring of data (with 25%-40% of individuals predicted to react to the first challenge dose with objective symptoms,^38^ which would result in a skewing of symptoms at lower doses toward more severe reactions). Our data are consistent with the findings of previous reports that anaphylaxis can occur in response to all levels of allergen:^10^^,^^43^, ^44^, ^45^ that is, individuals with peanut allergy who react to lower doses of allergen exposure are not at greater risk of severe reactions.

### Implications for clinical practice

Patients with a food allergy often report incidents of allergen exposure in response to which they experience only minimal (if any) symptoms and yet report significant symptom heterogeneity in response to a similar level of exposure in the past. In children, this might be interpreted as an indicator of natural resolution, although our data suggest that an inherent variability in reaction threshold (determined at FC) may be an alternative explanation. Increasingly, clinicians are advocating for the use of clinical thresholds (determined at FC) to inform the degree of dietary allergen avoidance required by any given patient (eg, whether to ignore precautionary allergen labeling on prepacked foods).^1^^,^^2^ However, a limitation of this approach has been the uncertainty relating to the reproducibility of clinical reaction thresholds over time.

In our analysis, 70% of individuals with peanut allergy demonstrated a relatively stable threshold, with any shift limited to a half-log change in clinical reactivity independent of the effect of any cofactors or changes in the food matrix into which the allergen is incorporated. This “intrinsic” shift in threshold followed a normal distribution, both for participants able to tolerate an ED_50_ exposure and for those with objective symptoms in response to ED_50_ (about 100 mg of peanut protein, which is equivalent to half a peanut). However, there is clearly potential for some individuals to demonstrate a far bigger change (up to 1000-fold) in threshold, which is important to consider with respect to individual patient advice. In children, a 1000-fold increase may imply natural resolution (which probably explains the mild skewing of data in some of the included cohorts), but we also observed a small number of individuals with a greater than 100-fold *decrease* in threshold. These observations are also important when considering the reproducibility of FC as a measure of efficacy for clinical intervention trials for food allergy, and they reinforce the need for randomization and the use of placebo intervention in clinical trials. In participants who react to low-level peanut exposures (eg, <50 mg of peanut protein), there can be up to a 10-fold increase in threshold of because of this intrinsic variability rather than because of a specific treatment effect (although a proportion of participants would be expected to show a fall in threshold for the same reason, given the normal distribution of data).

The inclusion of IPD also allowed for an assessment of reaction severity as well as *clinical sensitivity*, which is something that needs to be considered separately, given the uncertainty regarding the relationship between severity and sensitivity.^45^^,^^46^ Analogous to reaction thresholds for clinical symptoms, it has been proposed that patients also have a threshold for anaphylaxis; this may be at a threshold similar to or higher than that causing objective symptoms.^45^ Our data demonstrate that there is a similar degree of intrinsic variability in the amount of allergen needed to trigger anaphylaxis. Patients and clinicians should thus consider the fact that a history of anaphylaxis in response to a particular dose does not therefore imply that participants will *always* develop anaphylaxis in response to that level of exposure, although the controlled FC scenario does not represent a “community” exposure to allergen. In this analysis, 75% of those with anaphylaxis at initial FC did not develop anaphylaxis in response to the same (or lower) dose at subsequent exposure. Conversely, absence of anaphylaxis in response to a given dose of allergen cannot alone be interpreted as implying a low risk of anaphylaxis in response to that same dose on another occasion, particularly given the potential impact of cofactors.^30^ Reassuringly, however, very low-level allergen exposure (≤ED_05_) is considered, although approximately 5% of individuals who tolerated that dose on 1 occasion had objective symptoms on repeat exposure, none developed anaphylaxis.

### Strengths and limitations of this study

By limiting our analysis to peanut, we were able to undertake a robust analysis of data from more than 3000 FCs undertaken according to clinical trial protocols with predetermined objective criteria. Although this provides a large degree of confidence in the certainty of the estimates obtained at meta-analysis, it is likely that these data are also applicable to other food allergens. In support of this, 0.5% to 0.6% of individuals developed anaphylaxis in response to an approximately ED_05_ level of exposure in 2 prospective studies of patients with cow’s milk allergy.^43^^,^^47^ This rate was even lower for egg, wheat, and soy.^43^ We were unable to undertake sensitivity analyses based on participant age (as these data were not available because of data confidentiality regulations). Around 10% of the included data pertained to adult participants; thus, the analysis is skewed toward older children (with the vast majority aged 8-18 years). However, we did not identify any major differences between those studies (eg, TRACE) that included adults only. This is consistent with an analysis undertaken by the second Voluntary Incidental Trace Allergen Labeling (VITAL-2) expert panel, who reported that while the threshold distribution curves for adults and children with peanut allergy differ (with children being more sensitive than adults to peanut), this difference is not apparent for ED_05_ and ED_10_ estimates (ie, at the lower end of the dose-distribution curves).^48^

Despite variations in the inclusion characteristics of the included studies and specific challenge protocols, there was only a low-to-moderate level of heterogeneity observed at meta-analysis, providing reassurance as to the low level of uncertainty of the resulting pooled estimates. Furthermore, sensitivity analyses demonstrated that the heterogeneity was minimal when clearly defined criteria for LOAEL were applied.^37^ The sensitivity analyses demonstrated little impact on the overall pooled estimate, and arguably such impact might have resulted in a more conservative estimate because some studies (eg, TRACE study) used more rigid criteria to define dose-limiting symptoms, thus overestimating the rate of anaphylaxis at lower levels of allergen exposure. Similarly, the use of different anaphylaxis definitions across studies did not significantly affect the overall pooled estimates in sensitivity analyses.

Although differences in challenge protocols (including the interval between doses) can affect the apparent clinical thresholds,^37^^,^^49^ this would not have caused significant confounding in our analysis: first, the assessment of reproducibility was undertaken within a group of participants who underwent repeat challenge with the same team (and often with blinded clinicians) using an identical protocol (thus any confounder would be present at both challenges), and second, the meta-analysis took these differences in challenge protocols into account. In any event, the primary analysis (rate of anaphylaxis occurring in response to a low level of exposure) would not have been affected by differences in challenge protocols (such as duration in between doses), as in most studies, only the first 1 or 2 challenge doses would have been relevant. The skewing of the studies (toward more sensitive individuals, as evidenced by the rates of participants reacting to ED_05_ levels of exposure being greater than 5%) is an advantage because this increased the available data set and thus the power of the analysis. We do not believe that this would affect the estimate of anaphylaxis risk, because there is no reason to believe that the “lower-dose” reactors in the cohorts included in this analysis would have a rate of anaphylaxis in response that would differ from the rate among those in the wider population with peanut allergy. However, in an evaluation of the reproducibility of reaction thresholds, only participants who reacted to 1.44 g or less of peanut protein at baseline challenge were included, resulting in a degree of skewing toward lower-dose reactors. We cannot exclude the possibility that observer and/or subject bias might result in a lower threshold at the second repeat challenge (because of anticipation of a reaction). For example, in the TRACE study, there was a decrease in threshold with repeat challenge.^30^ This is evident in Fig 4. However, a skewing in the opposite direction (toward a reaction at a higher dose) was evident in some of the included studies of AIT. This might be due to a higher rate of natural resolution of peanut allergy in the relatively younger participants included in these studies or to inadvertent observer and/or subject bias due to anticipation of a positive effect from treatment in blinded studies of AIT. Overall, however, the data still followed a normal distribution, with no evidence of skewing in the pooled estimates. Although this analysis used data from studies that in most cases did not seek to address reproducibility, this does not negate the value of our analysis, given the size of the available data set and the use of IPD meta-analysis to generate pooled estimates.

Importantly, all of the FCs included would have been undertaken when the participants were well and without obvious cofactors that could affect reaction thresholds. Dua et al demonstrated that in the presence of cofactors, there was a drop in threshold in around 40% of participants, resulting in a small number of participants reacting to ED_01_ levels of exposure after having previously reacted to an ED_05_ amount.^30^ Such an effect needs to be considered when using threshold data to guide individual patient management or population-based risk management programs.

### Conclusions

In this analysis, around 5% of individuals reacting to an ED_01_ or ED_05_ level of exposure to peanut developed anaphylaxis in response to that dose. This equates to a risk of anaphylaxis in the broader population with peanut allergy of 1 and 6 per 2500 patients exposed to an ED_01_ or ED_05_ dose, respectively. This may be acceptable to consumers with a food allergy if the trade-off allows for an evidence-based approach to allergen risk management (eg, to decide on the need for precautionary allergen labeling)—particularly if those individuals at risk can be identified through the use of low, single-dose challenges. Although the reproducibility of reaction thresholds varies, less than 5% of individuals will react to a sub-ED_05_ level after having tolerated it previously, and those that do so are very unlikely to develop anaphylaxis. These data will assist regulators, public health agencies, and food business operators in establishing evidence-based approaches to allergen management as means to protect the consumer with a food allergy from accidental exposures.

Finally, this analysis highlights the significant value of combined data set analyses to more accurately define the characteristics of allergic reactions. We encourage our colleagues to share anonymized IPD generated by FCs to advance our understanding of food allergy.

**Clinical implications**

There is inherent variability in reaction thresholds at FC, but this does not adversely affect current attempts to improve allergen risk management for patients with food allergy.

## Figures and Tables

**Table E1:** Risk of bias in the included studies

Study∗	Design	Interval in between FC	Selection bias†	Attrition bias	Detection bias	Internal validity	External validity‡	Comments
Taylor et al, 2010^13^	Diagnostic	N/A	N/A	N/A	Low	+	++	No information on self-selection because of subjects declining to participate; challenge doses given every 15 min
Blom et al, 2013^14^	Diagnostic	N/A	N/A	N/A	Low	++	++	No information on self-selection because subjects declining to participate
Van Erp et al, 2013^17^	Diagnostic	N/A	N/A	N/A	Low	+	++	No information on self-selection because of subjects declining to participate; challenge doses given every 15-30 min
STOP-II 2014^21^	Interventional RCT	26 wk	Low	Low	Low	+	+	Group allocation not masked; 5-dose FC protocol
EuroPrevall 2015^4^^,^^5^	Diagnostic	N/A	N/A	N/A	Low	+	+	51 challenges eligible, data available for 43 subjects
Klemans et al, 2015^19^	Diagnostic	N/A	N/A	N/A	Low	+	++	No information on self-selection because of subjects declining to participate; challenge doses given every 15 min
Kukkonen et al, 2015^21^	Diagnostic	N/A	N/A	N/A	Low	+	+	No information on self-selection because of subjects declining to participate; inclusion cED ≤ 1255 mg, so some skewing of population
FAHF-2 2015^23^	Interventional RCT	N/A	Unclear	N/A	Low	+	+	Challenge doses given every 10-15 min
ARC001 2017^24^	Interventional RCT	20-36 wk	Low	Low	Low	++	+	Inclusion cED ≤ 143 mg, so skewing of population
VIPES 2017^25^	Interventional RCT	12 mo	Low	Low	Low	++	+	Inclusion cED ≤ 444 mg, so some skewing of population
TAKE-AWAY 2017^26^	Interventional RCT	N/A	Low	N/A	Low	+	–	No information on self-selection because of subjects declining to participate; inclusion cED ≤ 144 mg, so skewing of population
Purington et al, 2018^27^	Diagnostic	Median 2 years	Unclear	N/A	Low	+	–	Participants tolerating ≥500 mg excluded from analysis; 15-min intervals between challenge doses
PALISADE 2018^28^	Interventional RCT	12 mo	Low	Low	Low	++	+	Inclusion cED ≤ 144 mg, so skewing of population
PEPITES 2019^29^	Interventional RCT	12 mo	Low	Low	Low	++	+	Inclusion cED ≤ 444 mg, so some skewing of population
TRACE 2019^30^	Interventional RCT	3-9 mo	Low	Unclear	Unclear for repeat FC	+	+	Inclusion cED ≤ 1433 mg, so some skewing of population; cED significantly lower on open challenges conducted following initial DBPCFC.
BOPI 2019^31^	Interventional RCT	12 mo	Low	Low	Low	+	++	Group allocation not masked
POISED 2019^32^	Interventional RCT	2 y	Low	Low	Low	+	+	Inclusion criteria and requirement for cED ≤ 500 mg, so some skewing of population; challenge doses given at 15- to 60-min intervals
ARTEMIS 2020^33^	Interventional RCT	32 wk	Low	Low	Low	++	+	Inclusion cED ≤444 mg, so skewing of population
UMCG 2020^4^	Diagnostic	N/A	N/A	N/A	Low	+	++	

*cED*, Cumulative ED; *N/A*, not available; *RCT*, randomized controlled trial.

∗ Reference numbers after study authors or names refer to the reference list in the print article.

† Selection bias refers to possible differences in subject allocation between intervention and control groups, which was not relevant for studies that were not used for the IPD meta-analysis to assess reproducibility of reaction thresholds at DBPCFC.

‡ External validity assesses for the impact of participant selection bias and whether this affects whether the study data are generalizable to the overall population with peanut allergy. External validity is described as ++ (which means that all or most of the criteria have been fulfilled, and where not, the conclusions are very unlikely to alter), + (which means that some criteria have been fulfilled, and where not fulfilled or adequately described, the conclusions are unlikely to alter), and – (which means that few or no checklist criteria have been fulfilled).
